# Nanoencapsulation of Promising Bioactive Compounds to Improve Their Absorption, Stability, Functionality and the Appearance of the Final Food Products

**DOI:** 10.3390/molecules26061547

**Published:** 2021-03-11

**Authors:** Mirian Pateiro, Belén Gómez, Paulo E. S. Munekata, Francisco J. Barba, Predrag Putnik, Danijela Bursać Kovačević, José M. Lorenzo

**Affiliations:** 1 Centro Tecnológico de la Carne de Galicia, rúa Galicia n° 4, Parque Tecnológico de Galicia, 32900 San Cibrao das Viñas, Ourense, Spain; mirianpateiro@ceteca.net (M.P.); belengomez@ceteca.net (B.G.); paulosichetti@ceteca.net (P.E.S.M.); 2 Department of Preventive Medicine and Public Health, Food Science, Toxicology and Forensic Medicine, Faculty of Pharmacy, Universitat de València, Avda. Vicent Andrés Estellés, 46100 Burjassot, València, Spain; francisco.barba@uv.es; 3 Department of Food Technology, University North, Trg Dr. Žarka Dolinara 1, 48000 Koprivnica, Croatia; pputnik@alumni.uconn.edu; 4 Faculty of Food Technology and Biotechnology, University of Zagreb, Pierottijeva 6, 10000 Zagreb, Croatia; dbursac@pbf.hr; 5 Área de Tecnología de los Alimentos, Facultad de Ciencias de Ourense, Universidad de Vigo, 32004 Vigo, Ourense, Spain

**Keywords:** coating materials, antioxidants, antimicrobials, probiotics and prebiotics, essential oils, nanotechnology

## Abstract

The design of functional foods has grown recently as an answer to rising consumers’ concerns and demands for natural, nutritional and healthy food products. Nanoencapsulation is a technique based on enclosing a bioactive compound (BAC) in liquid, solid or gaseous states within a matrix or inert material for preserving the coated substance (food or flavor molecules/ingredients). Nanoencapsulation can improve stability of BACs, improving the regulation of their release at physiologically active sites. Regarding materials for food and nutraceutical applications, the most used are carbohydrate-, protein- or lipid-based alternatives such as chitosan, peptide–chitosan and β-lactoglobulin nanoparticles (NPs) or emulsion biopolymer complexes. On the other hand, the main BACs used in foods for health promoting, including antioxidants, antimicrobials, vitamins, probiotics and prebiotics and others (minerals, enzymes and flavoring compounds). Nanotechnology can also play notable role in the development of programmable food, an original futuristic concept promising the consumers to obtain high quality food of desired nutritive and sensory characteristics.

## 1. Introduction

Food manufacturing is facing a wide variety of challenges related to food safety and final appearance of the product, such as the control of certain chemical contaminants, microbiological problems or the obtaining of desirable physicochemical and organoleptic properties. To overcome these issues, nanotechnology arises to improve quality and safety of the final product.

Nanotechnology consists of the generation and utilization of materials, mechanisms or systems at a nanometer scale. Nanomaterials are commonly described as elements with a particle size below 100 nm, which have singular characteristics compared to their macroscale counterparts, mainly due to their large surface-to-volume ratios [1,2].

In food science, nanotechnology is an emerging area that is important for the future industrial productions, where newly developed properties from the particle reduction will probably provide improved sensory properties of foods (e.g., color, taste and texture). In addition, nanosubstances could be suitable for food protection while extending the storage [3,4]. According to Handford et al. [5], this technology offers new applications such as food packaging and the design of delivery systems for bioactive compounds (BACs). For this purpose, functional nanocapsules are developed with the aid of surfactants, reverse micelles and emulsion layers [6]. Additionally, their large surface area results in numerous advantages, such as good reactivity, aqueous solubility or efficient absorption, encapsulation by nanotechnology have obtained special and growing attention [7].

The process of encapsulation is based on enclosing a bioactive compound in liquid, solid or gaseous states within a matrix or inert material, usually a polymer. An enclosed wall can preserve the coated substance (food or flavor molecules/ingredients), increasing its stability in the corresponding environment. Encapsulation can promote greater stability of bioactives in foods with improved regulation of their release at the active physiological place [8,9]. Essentially, it has numerous of applications in science and industries, including cosmetics, medicals, pharmaceuticals and foods [10]. A common application consists of masking undesirable odors or flavors in food formulations, or simply changes liquid phases to solid ones [11]. Specifically, nanoencapsulation provides particles of <100 nm, and according to Jafari [11] and Nikmaram et al. [2], the encapsulated particles can be classified as nanocapsules (<0.2 μm), microcapsules (0.2–5000 μm) and macrocapsules (>5000 μm). One of the main benefits of applying nanoencapsulation is the homogeneity it imparts, resulting in better encapsulation efficiency and suitable physical and chemical properties. This process is an appealing alternative for the redesign of functional food components that can strongly influence the processability, bioavailability and stability of products [12,13].

Currently, more attention is given to the controlled discharge of bioactive raw materials under particular circumstances, thus leading to an extended shelf-life and ensuring the optimal uptake of nutrients [11,14,15]. In fact, encapsulation appears as one of the most encouraging methods for shielding delicate materials from undesirable circumstances or degradation due to oxidative conditions [7].

There are numerous crucial features that should be taken into account, as the final particle size, sufficient activity of ingredient, the form of discharge or the resistance of the capsules. Additionally, the wall material and economic issues are important for marketing of products produced with this novel method [11]. Moreover, a positive impact on the bioaccessibility and antioxidant activity in nanotechnology-based delivery systems has been recently observed [16]. Bioaccessibility (ability of the digestion to free-nutritive compound from foods) must be the first step to evaluate their physiological absorption [17,18]. Therefore, bioaccessibility assessments are needed to validate functional foods and estimate the ability of specific BACs to deliver positive effects on health.

This review aims to highlight basic and applied aspects of encapsulation processes in nanotechnology, the characteristics that condition the efficient use of NPs, the main coating materials and BACs and ingredients encapsulated and their application in food systems.

## 2. Applications and Technological Advances of Nanoscience in the Food Industry

In recent times, nanotechnology is getting more and more attention from food and health care sectors due to the large number of benefits it offers [19]. As aforesaid, including enhanced bioavailability and reduced levels of BACs, the capacity for directed delivery of bioactive substances to particular tissues or organs is also improved [15]. Both the minimum particle size and a significant surface area give nanostructured materials their exceptional properties and capabilities for their application in the food industry. Although, in comparison to other fields, nanotechnology in food science only began to address its application that could provide important benefits for global society. Major improvements have been focused on modifying the texture of food products, encapsulating edible substances or additives, elaborating original flavors and improving the bioaccessibility/bioavailability of dietary components [6]. Recently, Ponce et al. [6] stated that there is evidence to declare that the present direction of nanotechnology studies in food sector cover five general areas: (1) food manufacturing, (2) packaging, (3) safety and quality, (4) nutraceuticals and (5) functional foods.

**Figure 1:**
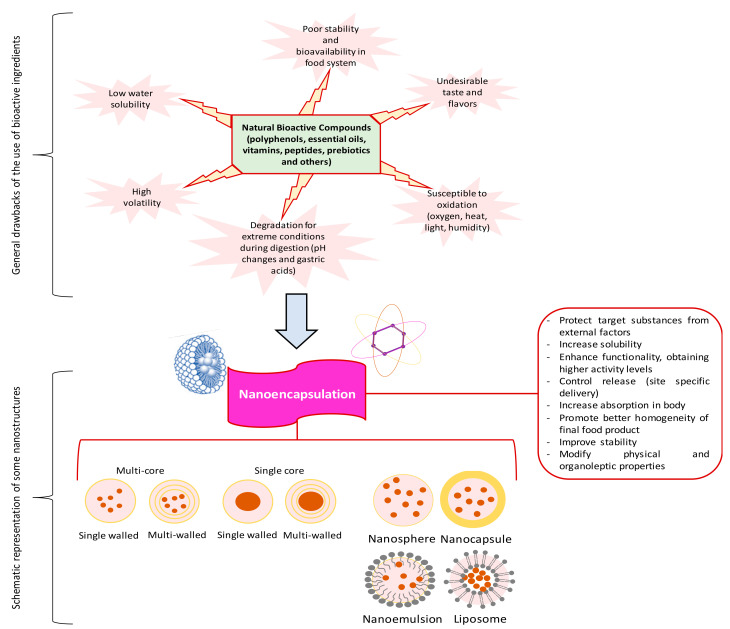
Limitations of the use of bioactive compounds (BACs) in the food industry and advantages of nanoencapsulation to enhance their applicability.

Among the greatest benefits of nanotechnology for food products are [11,15]: (1) improved stability and shelf-life of BACs, preserving them against degradation during processing, distribution and storage; (2) improved mechanical and thermal properties; (3) integration of food additives and functional raw materials, e.g., ω- 3 fatty acids; (4) mask undesirable tastes; (5) protect nutraceuticals from extreme conditions as low gastric pH and optimize their release during the digestion process; (6) increase the solubility of functional constituents while strengthening their dietary value; (7) obtain higher activity levels of the encapsulated ingredients (antioxidants, antimicrobials, probiotics, etc.); (8) change the flavor and texture of food components; (9) develop optically transparent beverages (nanoemulsions that have oil droplets <100 nm); (10) produce low-fat or low-calorie food products and (11) induce better homogeneity of the food system (Figure 1).

## 3. Classification of Nanoencapsulation Systems/Techniques

Briefly, nanoencapsulation consists of coating target compounds (internal phase or core substance; Table 1) with different external materials (generally known as shell, wall or carrier material; Table 2) to generate small particles that may exert positive or healthy effects.

Traditionally, nanoencapsulation technologies have been divided into two general mechanisms for the elaboration of nanomaterials: “top-down” and “bottom-up”. According to the “top-down” technique, particle size is decreased with encapsulation by the application of accurate tools. On the other hand, the “bottom-up” method has the particle size increased. Techniques such as self-assembly and self-organization of molecules, supercritical-fluids, coacervation, inclusion complexation and nanoprecipitation are considered bottom-up methods, while emulsification is a top-down approach. In some cases, both approaches can be combined to yield optimal results. This classification is not used currently [11], but nanoencapsulated systems tend to be separated into liquid–liquid, solid–liquid and solid–solid systems. The main liquid–liquid process, which is usually employed for solubilization, is oil–water (O/W) emulsion-based system [20].

A variety of nanoencapsulation processes for food BACs or nutraceuticals have been performed in the last years. For instance, methods resembling nanostructured lipid carriers (NLCs), nanoemulsions, nanosuspensions, nanoliposomes, biopolymer nanoparticles (NPs) or micelles made of polysaccharides and/or proteins have been developed [11]. Panigrahi et al. [21] described the liquid-based processes (nanoprecipitation), emulsification–solvent evaporation, coacervation, ionotropic gelation, supercritical fluid extraction of emulsion (O/W) and electrochemical-based techniques (electrospinning and electrospraying) as suitable techniques for nanoencapsulation.

Furthermore, nanostructures can be also organized into two basic classes: single-core and multicore. Particularly, in the case of nanocarriers, two mechanisms of nanosphere and nanocapsule have been described by Jafari [11]. This author classified nanoencapsulation techniques into five groups, which takes into account the main procedure/component used to develop the nanocapsules, including: (1) lipid formulations (nanoemulsions, nanoliposomes and nanostructured lipid carriers); (2) natural nanocarriers (caseins, cyclodextrins, nanocrystals and amylose nanostructures); (3) specialized equipment (electrospun nanofibers, electrosprayed NPs and nanospray dried particles); (4) functional biopolymeric NPs and (5) disparate techniques (protein nanotubes and carbohydrate nanogels).

The benefits offered by these nanodelivery systems depend on the compatibility of NPs properties with the properties of the BACs and the desired application [22], which allow their usage in a wide diversity of solid foods and beverages [6]. It is essential to take into account several parameters for optimal NP preparations, including pH, selection of organic solvents, processing time, type of polymer(s), presence of surfactants, target compound(s)-to-polymer(s) ratio and method(s) of drying [15]. In fact, NPs resulting from these encapsulation methods usually vary in size, shape, mechanical properties and composition (Table 3), which are going to condition their absorption, distribution, metabolism and excretion [11,15].

Particle size is considered the primary characteristic, which would determine the functional properties of the nanoencapsulated product [23]. A further increase in polymer concentration and/or double-walled NPs gave rise to an increase in the particle size but also to a change in the morphology [24]. In this way, smaller particle size leads to better dissolution behavior, probably due to the increase in surface area to volume ratio of NPs. Moreover, polydispersity index (PDI) is also an important factor that indicates size distribution. It has been established that values higher than 0.6 are regarded to indicate polydispersity, while values under 0.3 are considered to indicate monodispersity. Therefore, this parameter could indicate that size of NPs was or not distributed uniformly in suspensions [25].

In the case of zeta potential (ZP), which establishes the potential differences between the immobilized and dynamic ionic layers on the charged particles, plays an important role in the stability of NPs [26]. In general, NPs with ZP more positive than +30 mV or more negative than −30 mV lead to more stable complexes [27]. In addition to these parameters, ionic strength, pH, type and concentration of coating materials, and the ratio between them, are effective on the amount of surface charges and electrophoretic mobility resulting in variable ZPs of complexes [26].

**Table 3:** Effect of NPs characteristics on their stability and potential applications in the food sector.

Active Compound	NPs	Encapsulation Conditions	Physical Properties	Outcomes	Ref.
Curcumin	Zein LMW 25–30 kDa	Electrospray/electrohydrodynamic atomization technique Zein concentration: 2.5% (*w/w*) Flow rate: 0.15 mL/h Voltage: 14 kV Working distance: 7 cmCurcumin: Zein ratios: 1:500, 1:100, 1:50, 1:20 and 1:10 (*w/w*)	NPS: 175–250 nm EE: 85–90%	Zein compact NPs charged with curcumin, making possible to extend the use of curcumin like a coloring agent in aqueous food products.	[28]
DHBA, PA, and RA	Chitosan: LMW: 107 kDa, 0.2% (*w/v*) HMW: 624 kDa, 0.4% (*w/v*)	Ionic gelation RA 0.6% (*w/v*); PA 0.3% (*w/v*); DHBA 0.3% (*w/v*)	LMW and HMW showed, respectively: NPS: 356–374 nm and 511–604.2 nm PDI: 0.22–0.23 and 0.15–0.20 ZP: 28–30 mV and 22.9–31.1 mV pH: 5.0–5.7 and 5.2–5.5	NPs produced with HMW chitosan produced higher values of inhibition. *E. coli* and *B. cereus* were the most susceptible to NPs.	[29]
D-limonene	Pectin-whey protein nanocomplexes	Whey proteins (4, 6 and 8% *w/w*) Pectin (0.5, 0.75 and 1% *w/w*)	Spherical NPs NPS: 160 nm EE: 88% ZP: −0.53 mV pH = 3	The resulted NPs can be used in food products such as cakes, muffins, biscuits and juices. These NPs protected flavoring during processing and storage, and also its release can be controlled.	[26]
Epigallocatechin gallate	Chitosan/ β-lactoglobulin NPs	EG (0, 10, 20, 30, and 40 mg) Chitosan (1.0–2.0 mg/mL) β-Lg (0, 10, 20, 30, and 40 mg/mL)	NPS: 193.8–289.2 nm EE: 40–60% PDI: 0.13–0.21 ZP: 28.3–24.9 mV pH 6.0–7.0	Double-walled chitosan/β-Lg NPs. Prolonged release capabilities and excellent adhesion properties of could enhance the effective absorption of these NPs in the human intestine.	[24]
Eugenol	Zein-caseinate-pectin complex	Spray drying	NPS: 140 nm pH 6.6	NPs with spherical shape. Caseinate allowed it to stabilize complex NPs during spray drying and storage.	[30]
Guabiroba fruit	PLGA	PLGA-lactic: glycolic acid ratio: 65:35 or 50:50 (MW 40,000–75,000 g/mol)	NPS: 202.5–243.8 nm PDI: 0.37–0.43	PLGA NPs can be used as a delivery system for phenolic compounds at levels lower than originally required for enhanced functional properties.	[31]
Quercetin	Chitosan-alginate	NP_1_: Sodium alginate (3 mg/mL); calcium chloride (3.35 mg/mL); chitosan (0.75 mg/mL). NP_2_: Chitosan solution (3 mg/mL); calcium chloride; sodium alginate (0.75 mg/mL)	NPS: ≅400–600 nm ZP: −30 to + 30 mV	NPs prepared with higher concentration of sodium alginate displayed smaller size and negative values for ZP. NPs showed low or absent toxicity in different cell models in vitro and better protection against oxidative stress than free quercetin.	[32]
Thymol	Zein stabilized with sodium caseinate and chitosan hydrochloride	Thymol: zein ratios: 1:1, 1:2, 1:4, 2:1	NPS: ≅520 nm EE: 80.51–82.89% PDI: 0.16–0.24 IP: 6.18 to 5.05 ZP: 64.45 mV pH ≅4.3	Increase of EE.	[33]

β-Lg: β-lactoglobulin; DHBA: 2,5-dihydroxybenzoic acid; EE: Encapsulation efficiency; EG: Epigallocatechin gallate; HMW: High molecular weight; IP: Isoelectric point; LMW: Low molecular weight; NPs: Nanoparticles; NPS: Nanoparticle size; PA: Protocatechuic acid; PDI: Polydispersity index; PLGA: Poly(D,L-lactic-co-glycolic) acid; RA: Rosmarinic acid; ZP: Zeta potential.

Micelles have been applied to raise the solubility of bioactive components, such as vitamins, enzymes, β-carotene, soy isoflavones or ω-3 fatty acids. A nanotechnology-based carrier system containing 30 nm micelles was created by a German company Aquanova (Darmstadt, Germany) to encapsulate bioactive substances. The nanoscale carrier system, called NovaSol^®^, improves the bioavailability and potency of the target compounds [34]. In the meantime, the development of nanoliposomes has been described as interesting for food technologists due to the attractive opportunities it offers in a variety of areas (encapsulation, controlled release, increased stability and bioavailability). Nanoliposomes were applied in the food industry to provide flavors and, lately, their capacity has been examined to integrate nutraceuticals, nutrients, enzymes or food additives and food antimicrobials [35].

Another suitable method for the generation of steady nanosized particles is ionic gelation, a simple, mild and solvent-free technique. This alternative approach consists of the interaction between positively charged polymers, as chitosan and polyanions. This results with the formation of inter- and intramolecular cross-linkages without employing toxic agents or high temperatures [36]. However, ionic gelation according to Kunjachan et al. [37], as earlier known as ion-induced gelation, leads to NPs and microparticles with some shortcomings. These include inappropriate surface morphology, high dispersibility index, delicate particulate mechanism and absence of proper surface modification spaces to connect functional moieties.

On the other hand, relatively new electrospinning techniques for food science, received notable attention over the last years due to simplicity, versatility and effectiveness [38]. In the same sense, nanofibers formed by electrospinning have numerous structural and functional benefits, for instance a large surface-to-volume ratio owed to large porosity, encapsulation efficiency (EE) (without thermal energy) and the better stability of the final encapsulated ingredients [39]. Similarly, electrospraying or electrohydrodynamic atomization is an original and simple approach based on handling the high electrostatic potential. In particular, electrospraying has been reported as suitable for encapsulation of heat sensitive food ingredients due to the nonthermal nature of this method [40].

**Figure 2:**
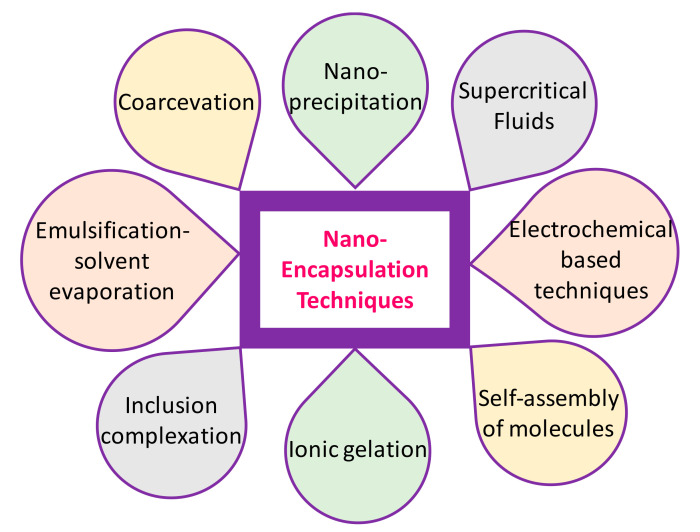
Techniques for nanoencapsulation of food bioactives.

Finally, Pisoschi et al. [41] compiled a specific list of nanostructure and nanoencapsulation techniques that are adequate for antioxidants and antimicrobials as: (1) association micelles-based nanoencapsulation; (2) lipid-based nanoencapsulation (nanoemulsions, nanoliposomes and solid lipid NP incorporation); (3) encapsulation based on biologically-derived and non-biological polymeric nanocarriers (coacervation, ionic gelation, electrostatic complexation, incorporation in layer-by-layer deposited nanolaminates, emulsification-solvent evaporation, nanoprecipitation and supercritical fluid technique); (4) cyclodextrin-based nanocapsule incorporation-molecular inclusion; (5) nanofiber encapsulation-electrospraying and electrospinning; (6) carbon nanotube and nanocomposite encapsulation; (7) drying techniques that follow some of the nanoencapsulation techniques (freeze-drying and spray-drying) and (8) encapsulation in specialized nanostructures (Figure 2).

## 4. Coating Materials

Choice of adequate coating materials for particular purpose is vital for encapsulation. A number of parameters should be evaluated prior designing encapsulation system, e.g., the particle size, the final physical state, safety and environmental concerns and the economic issues. Regarding the function of the different ingredients, the wall material should be selected in order to satisfy specific target requirements [11]. The coating material should be inert with respect to the active ingredients, stabilizer of the core compound, easily soluble, allow controlled release, be compatible with processing conditions (pH/temperature) and core substances [41]. More specifically, the features of an adequate shell include: (1) stabilization and preservation the core material against humidity, oxygen and light; (2) promote controlled diffusion under specific conditions; (3) inert with the BACs; (4) be food-grade and containing no adverse tastes and flavors; (5) present film-forming ability, low viscosity and high solubility and (6) low cost.

Generally, nanocarriers are divided into organic-based (polymeric and lipid-based NPs such as nanoemulsions or liposomes), inorganic-based (including metallic nanostructures, e.g., quantum dots), or a combination of both [4]. Numerous natural and synthetic polymers are suitable for electrospraying/electrospinning processes. Nevertheless, only “generally recognized as safe” (GRAS) components are permitted for foods [40].

Despite nanoencapsulation offers a large number of benefits there is increased awareness about the possible negative side effects, since some materials can be harmful to human health and, in fact, some substances that are well known and previously used in diverse products present new potential risks due to fact that nanomaterials have novel properties and different molecular and atomic dimensions. The most appropriate nanoscale carrier materials for food applications are carbohydrate-, protein- or lipid-based alternatives [18]. According to Malekhosseini et al. [8], numerous carries have been used for delivery of nutraceutical compounds, such as chitosan, peptide–chitosan and β-lactoglobulin NPs or O/W emulsion biopolymer complexes.

### 4.1. Natural Polymers Used for the Delivery of Bioactive Ingredients

Today the trend is towards the use of biodegradable polymeric NPs due to their favorable properties such as good biocompatibility, easy design and preparation, structure variations and interesting biomimetic characters [42]. These natural biopolymers are produced by controlled assembly of two different biopolymers such as protein and polysaccharide molecules. In addition, the protection of bioactive ingredients by nanocomplexes production through electrostatic attractions between negatively charged polysaccharides and positive proteins emerges as a novel and promising technology, since provides higher stability of NPs. Therefore, the polysaccharide–protein nanostructures are more interesting than pure single biopolymer NPs. Substances as chitosan, cellulose, maltodextrins, sodium alginate, gelatin, albumin, zein or whey protein are biopolymers that have been extensively studied for food encapsulation [15,40]. Particularly, milk proteins, mostly from whey, have been widely considered in the investigations of polysaccharide–protein complex systems [14]. In a current study, whey proteins (4%, 6% and 8% *w/w*) and pectin (0.5%, 0.75% and 1% *w/w*) were used to nanoencapsulate d-limonene at different pH values (3, 6 and 9) [26]. The results suggested that these wall materials would be able to nanoencapsulate (EE of 88%) appreciable amounts of D-limonene at high stability (83.0–98.5%).

#### 4.1.1. Carbohydrates

The most commonly used carbohydrates are chitosan, pectins, maltodextrins, corn syrup solids, acacia gums (gum Arabic), starch, maltodextrins and β-cyclodextrins. They have important properties, e.g., non-toxicity, good stability, biodegradability and bioadhesibility, which promote their application as delivery systems [43]. In particular, their thermal stability makes them suitable carriers for protecting thermolabile compounds during food processing under high temperatures, hence acting as an interesting option that is superior to lipid- or protein-based materials [44]. Frequently these polysaccharide-based delivery systems are classified by their origin as: herbal (e.g., pectin, starch or cellulose), algal (e.g., alginate and carrageenan), microbial (e.g., dextran and xanthan gum) and animal (e.g., chitosan) sources [43].

Walls made of chitosan have several advantages for nanocapsules synthesis, such as controlled release, use of safe organic solvents, and simple application in comparison to other polymers [9]. Specifically, the one formed from the total or partial deacetylation of chitin has been approved as GRAS, and recently it is receiving much attention for its use in the encapsulation of bioactive ingredients and nutraceuticals [45]. Mainly, due to non-toxicity, antimicrobial properties, biocompatibility, stability, biodegradability and its ability to develop films and particles [7,36,46]. Chitosan with sodium alginate were efficiently used to nanoencapsulate bioactive substances extracted from Egyptian prickly pears for application in guava juice [13], since it allowed the improvement of the antioxidant activity of the BACs at the same time that it enhanced their quality and stability. The NPs formulated with chitosan–sodium alginate and with chitosan were those showing the best results. Chitosan can also be efficiently nanoencapsulated in combination with γ-poly glutamic acid to improve the antimicrobial activity of rosemary (*Rosmarinus officinalis* L.) extract characterized by being poorly soluble [47]. The combination of the recognized properties of this plant as a significant source of antimicrobial and antioxidant compounds (mainly attributed to carnosic acid and carnosol) and the improvement of stability and solubility achieved through nanoencapsulation resulted in a successful application as antimicrobial NPs in beverages. This is demonstrated by the reduction in the microbial counts of *Bacillus subtilis* (0.5–3.6 log CFU/mL) in a ready-to-drink barley tea beverage. This reduction was size dependent, so that highest activity was observed for the largest particles (no counts vs. <4.0 log CFU/mL and ≅6 log CFU/mL for 600.6 nm, 212.2 nm and control samples, respectively).

Pectin is a complex macromolecule in nature, and main structural element in plant’s cell walls. It can be used as raw material for the buildup of galacturonic acid as its main chain (homogalacturonan) consists of galacturonic acid units, with α-D connector (1–4) esterified with methyl groups and/or acetylated [48]. Rehman et al. [49] recently reviewed the perspective of pectin as a carrier in different nanoencapsulation techniques (nanocomplex, nanohydrogel, nanoliposomes and nanoemulsion), with a focus on its applications for encapsulating natural bioactive ingredients. The authors reflected that pectin is a promising carrier for nanoencapsulation of bioactive ingredients such as vitamins, saffron and D-limonene, allowing its controlled release in the body. In addition, its use as complex with proteins and lipids allows to improves its beneficial effects in terms of stability and controlled release rate. In this regard, Lopes et al. [50] found that nanoliposomes coated with pectin or polygalacturonic acid can be used for lysozyme and nisin encapsulation, controlling effectively bacterial pathogens in a real food system such as Ultra-High Temperature (UHT) milk. Mohammadi et al. [51] achieved the nanoemulsion of olive leaf phenolic compounds with a whey protein concentrate–pectin complexes. The results were satisfactory since a high stability (96.64%) and slow controlled release (8.1%) of the encapsulated compounds by transport of the entire inner droplets were obtained. The results showed that nanoencapsulation technique around oil particles have a significant influence on the release of inner components. Moreover, double layers had more elasticity and aggregations than single-layers, resulting in a high stability [52]. Positive effects were also found when citrus peel pectin was combined with bovine serum albumin to form a nanohydrogel with vitamin C [53]. As a result, a stable food system with an EE of 65.31%, an enhanced vitamin C retention (73.95%) and a release in two distinct stages (burst release and sustained release) were obtained.

Cyclodextrins and its derivatives have been recommended as food additives with a variety of purposes, including the protection of lipophilic compounds, being able to improve the solubility of food colorings and vitamins, stabilize flavors, essential oils and other edible constituents, and to mask undesirable tastes or odors. Among cyclodextrins, β-cyclodextrin is the most frequently used. Recently, Quilaqueo et al. [18] reported the convenience of using β-cyclodextrin complexes to protect the bioactive properties of piperine, a beneficial nitrogenous compound with poor solubility in water, increasing its bioaccessibility and its in vitro antioxidant capacity along the gastrointestinal tract. The higher ratio β-cyclodextrin complexes: piperine (4:1) allowed it to obtain the highest loading efficiency and release in the colon. In contrast, despite the fact that nanoencapsulation is associated with masking some types of unpleasant odors and tastes [54], in this case it was not able to mask he pungent flavors. In the same way, the nanoencapsulation of lutein with hydroxypropyl methyl cellulose phthalate protected this functional food component, thus improving the application of lutein in the food industry [55].

#### 4.1.2. Proteins

Proteins are biological polymers made of amino acids connected together by peptide bonds. There are diverse amino acids that are arranged according to their features in the side groups as aliphatic, aromatic, charged (positive or negative) or polar. Proteins widely used in food manufacturing are extracted from herbal (soy and zein) and animal (casein, egg and whey protein) sources or by hydrolysis. Proteins have significant dietary value and abundant functional properties, for example gelation, emulsification or water binding capacity. Due to their versatility, a variety of structures can be generated (particles, films, fibers, tubules and hydrogels), offering the opportunity for delivering hydrophobic or hydrophilic bioactive components [43]. Additionally, in general, proteins have been catalogued as a natural, biodegradable and safe delivery systems.

Both, single or a combination of different proteins can be applicable to nanodelivery structures. Fathi et al. [56] reviewed protein-based nanoencapsulation approaches, including the physicochemical characteristics. Protein modification processes were useful to extend the functionality and behavior of bioactive agents in different protein-based nanocarriers, hence it is the main candidate for the formation of nanoparticles, nanogels and nanofibers from different origins. In this sense, Balandrán-Quintana et al. [57] obtained NPs from α-lactalbumin with an average diameter of 404 nm and an average inner diameter of 71 nm, while Monteiro et al. [58] mixed two proteins (α-lactalbumin and lysozyme) to produce micro- and nanostructures.

Whey, sodium caseinate and soy protein isolates have been used routinely for encapsulation of target compounds. Casein micelles have been suggested as appropriate carriers for different active ingredients, such as vitamin D3, curcumin, polyphenols, α-tocopherol, quercetin or polyunsaturated fatty acids [8]. Curcumin is a natural chemical compound found in turmeric (*Curcuma longa*), which has been related with numerous health benefits, including inhibition of cancer cell proliferation, antitumor activity, antibiotic (against bacteria and viruses), antioxidant potential, decrease of ageing, anti-inflammatory capacity and antitumor activities [59]. Despite its promising medicinal utilities, the efficient and wide application of curcumin have been limited owning to its low bioavailability derived from a high hydrophobicity, poor aqueous solubility, poor absorption, low stability and rapid metabolism. In this sense, nanoencapsulation can be a useful technique to avoid the technological obstacles and to improve the conservation of this functional compound and its delivery [15]. In this regard, a recent study developed casein NPs of sodium caseinate with curcumin or quercetin by hierarchical approach (ligand binding to sodium caseinate and then either reassembling the micellar nanostructures or formation casein NPs) [60]. The developed system displayed a high entrapment efficiency (>90%) and bioprotection for these hydrophobic nutraceuticals into fat-free clear beverages.

In the case of whey protein concentrate, different globular proteins are included, mainly β-lactoglobulin and α-lactalbumin. Moreover, it is characterized for incorporating hydrophilic and hydrophobic chains, and both with charged and uncharged amino acids [26]. The encapsulation by desolvation using β-lactoglobulin was used to nanoencapsulate mangiferin, a bioactive compound with different therapeutic activities that was obtained from *Curcuma amada*. The developed NPs can be stored for 30 days at 4 °C retaining its activity. In general, the investigation reported that they might be a promising candidate for oral delivery without toxic consequences [61]. The combination whey protein: curcumin could be also used as safe alternatives to synthetic polymer and may improve pharmacokinetic properties of curcumin. In this regard, Jayaprakasha et al. [62] demonstrated that the encapsulation of curcumin with whey protein would enhance the inhibition of colon cancer cells’ proliferation.

#### 4.1.3. Zein Nanostructures

Zein is a proline-rich storage protein from corn commonly utilized in food industry, which is characterized by a ratio of three quarters hydrophobic and one quarter hydrophilic amino acid units, which results in a lack of solubility in aqueous media. In contrast, it presents good solubility in various organic solvents, such as ethanol. Due to this, zein is a promising building block for preparing encapsulation and delivery systems for water-insoluble compounds, such as ω-3 fatty acids, essential oils, vitamin D3, resveratrol, quercetin, curcumin and lutein [55]. For instance, Wu et al. [63] reported that the retention of essential oils within zein nanostructure enables their dispersion in water with improvement of their antimicrobial and antioxidant capacity. According to Sozer and Kokini [64], zein nanostructures have the ability to constitute a tubular network. Moreover, it also shows other benefits as good elasticity or being renewable and biodegradable [39].

All this makes zein has been widely used in the food industry as a coating material [39]. In this regard, there are some studies about the electrospinning of zein in the food science, especially by inclusion functional compounds inside them. Dai et al. [65] investigated the formation mechanism, structural characterization and stability of curcumin in zein-lecithin composite NPs formed by antisolvent coprecipitation. Authors stated that these NPs could be a potential carrier for water-insoluble bioactive components with enhanced EE (99.83%) and chemical stability.

### 4.2. Fat and Waxes

Commonly, lipids are known as oils or fats depending on their physical form at room temperature, e.g., liquid or solid state. Furthermore, these substances are organized as nonpolar (cholesterol or triacylglycerol) or polar (phospholipids) compounds, which leads to important variations in their solubility and functional properties. Lipid-based delivery systems possess several advantages including possibility of trapping BACs with different solubility. In particular, instable molecules with high hydrophobicity can be preserved from degradation, thus providing their better stability during storage [43].

Some examples of lipid carries are bees wax, vegetable oils, lecithin and medium chain triglycerides [11]. Akhavan et al. [66] summarized the most recent nanoencapsulation developments incorporating formulations that are mainly based on lipid components. Authors concluded that nanocapsules containing lipids are interesting alternatives to other carriers due to a series of features they offer. They include improved solubility, high bioavailability and a controlled release of the nanoencapsulated food active ingredients. Likewise, Kiani et al. [67] described lipids as suitable carriers to achieve a good solubility, stability and efficacy of BACs. In fact, these authors produced novel form of vitamin D3 loaded in lipid nanocapsules by phase inversion method, and after a sensory evaluation, proved their potential application for development of fortified milk.

*Humulus lupulus* L. (Hop) is a perennial and climbing plant from the family of Cannabaceae, being flavonoids, humulones (α-acids) and lupulones (β-acids) the main components of hop extract. Lupulon is the main component of β-acids that along with the xanthohumol are used to substitute synthetic additives in food products. In this way, lupulon–xanthohumol loaded nanoliposomes were incorporated as promising nitrite replacer in cooked beef-sausage. In addition to preventing microbial growth and lipid oxidation, nanoencapsulation allowed it to mask the associated bitter taste, avoiding adverse effects on sensory properties [68].

## 5. Nanoencapsulation of Bioactive Compounds and Food Ingredients

**Figure 3:**
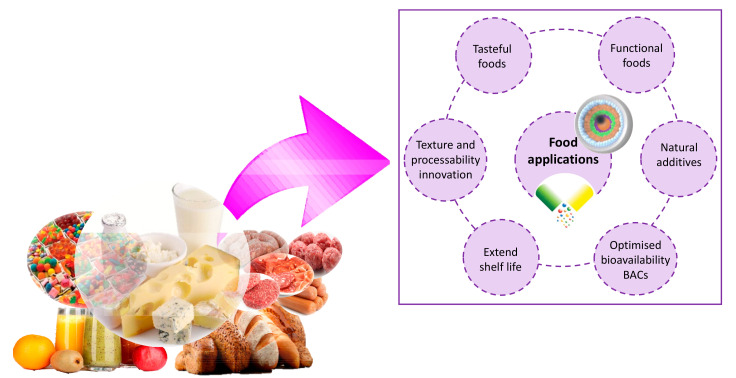
Improvement of stability, functionality and appearance of food products through nanoencapsulation of BACs.

There are numerous BACs, other hydrophobic or poorly water-soluble nutrients important for human diet and essential for maintaining good health and well-being of consumers. For instance, essential fatty acids, carotenoids, phenolic compounds and insoluble vitamins are included in this category. The principal challenge for the incorporation of these substances in the pharmaceutical and food industries was to overcome their low solubility and bioavailability (Figure 3).

In this sense, nanoencapsulation could be an encouraging method for shielding many food constituents against certain physiological circumstances or degradation with masking some types of unpleasant odors and tastes [54]. Reducing the particle size of BACs may enhance their stability, bioavailability, solubility and delivery, thus promoting their functional activity. In fact, nanotechnology has already been successfully used to solubilize and disperse lipophilic components in water and fruit beverages (carotenoids, phytosterols and antioxidants). In addition, nanoencapsulation can control the release of active ingredients (phenolic compounds, proteins, enzymes, vitamins, minerals and others) and improve the characteristics of the final food product [6,69]. de Souza Simões et al. [43] discussed the main BACs that have been used in foods for health promoting, including antioxidants, antimicrobials, vitamins, probiotics and prebiotics and others (minerals, enzymes and flavoring compounds) (Table 1).

### 5.1. Phenolic Compounds and Carotenoids

Polyphenols comprise a considerable diverse group of molecules, which include phenolic acids, flavonoids and many others. They have gained great interest in food production from both, society and industry. These compounds are widely recognized for their antioxidant capacity and interesting health benefits, including anti-inflammatory effects and the prevention of diet-related chronic disorders such as cardiovascular diseases, neurodegenerative diseases, type 2 diabetes and some cancers [31,70]. They commonly exert significant antioxidant properties by scavenging radical oxygen species, but are unstable in the presence of light, oxygen and heat during processing and storage [71]. In addition, they show low bioavailability, so their application can be limited due to their instability during digestion, because of pH, enzymes, interaction with other nutrients, etc. [31]. Furthermore, various phenolics such as flavanols and flavanones present a bitter taste that limits their use in the food production [69,72].

The encapsulation of these compounds with nanotechnology has been suggested to address most of these issues and to improve functional properties of foods (Table 4) [31]. For instance, a phenolic extract from guabiroba fruit was nanoencapsulated in poly (d,l-lactic-co-glycolic) acid using the adapted emulsion–evaporation procedure, thus preserving phenolic content and its bioactivity until consumption, and for a longer release time [31]. Guan et al. [73] nanoencapsulated caffeic acid phenethyl ester (one of the major hydrophobic bioactive components of propolis extract) in aqueous propylene glycol using sucrose fatty acid ester and a temperature-cycle method to achieve improved physiological properties and activities against cancer cells.

Regarding the development of functional foods, Tang et al. [84] used chitosan and poly (γ-glutamic acid) to produce nanostructures for oral delivery of tea catechins, which can be applied as food additives for drinks, foods and dietary supplements. These polyphenolic bioactives are responsible for most of the favorable health effects (antioxidative, anti-inflammatory, anticarcinogenic or antihypertensive) of green tea extracts [85]. However, their bioefficacy will depend on their bioavailability [86]. This complex process, which involves several phases (liberation, absorption, distribution, metabolism and elimination) is relatively poor for catechins [87]. Puligundla et al. [87] discussed the advances related to improvement of in vitro and in vivo bioavailability of green tea polyphenols using nanotechnology approaches. The nanoencapsulation in the form of biopolymer-based NPs, is the most popular approach to enhance their intestinal permeability and absorption. In addition, nanoencapsulation would also avoid the potential antinutritional properties of tea polyphenols.

Another compound, with increased scientific and industrial interest, is aspalathin that exerts particular capacity to improve glucose and lipid metabolism. This substance can be found in high amounts in *Aspalathus linearis*, traditionally used for the production of rooibos tea. Nevertheless, similar to curcumin, aspalathin has poor bioavailability and peculiar structure that limits its intestinal absorption with high sensitivity to oxidation. For this reason, Human et al. [12] investigated the production of NPs of aspalathin by electrospraying using both natural (chitosan and lecithin) and synthetic biodegradable esters (poly(lactide-co-glycolide) (PLGA) and Eudragit S100^®^ (methacrylate acid copolymer, ES100) polymers, comparing the results with those found with conventional methods. The most favorable NPs characteristics were obtained using synthetic polymers and electrospraying method (55.4% and 50.8% vs. 1.6% and 1.2% for ES100 and PLGA in electrospraying and conventional methods, respectively). On the contrary, natural polymers reflected higher efficiency with conventional methods (31.1% and 86.8% vs. 4.4% and 74.4% for chitosan and lecithin, respectively). To sum up, high EE (55.4%) and LC (12.7%), small particle size (189.9 nm), high ZP (−44.3 mV) and a controlled slow-release profile were shown by ES100.

In addition to their beneficial properties on health, polyphenols also had important technological applications. This is the case the chlorogenic acids present in green coffee beans. These compounds had the ability to interact with proteins of foods, contributing to the formation of some specific aroma volatiles. Budryn et al. developed potential nanocomplexes between chlorogenic acids and β-cyclodextrin to supplement the aroma volatile profile of products as bread, cookies, caramel cottage cheese, nutty filling and mushroom or meat stuffing [74]. In the same way, curcumin and lutein NPs have been developed to increase the solubility of these natural substances and enhance their technologic applications.

Additionally, various researchers documented significant phenolic contents from olive leaves with emphasis on tyrosol, oleuropein, hydroxytyrosol, rutin and verbascoside [88]. Tavakoli et al. achieve the nanoencapsulation of oleuropein with a considerable EE through lecithin-cholesterol system (70.7–88.2%) [78]. The incorporation of oleuropein nanoliposomes in yogurt allowed it to minimize the syneresis rate and improved the antioxidant activity, contributing to good sensory attributes.

Novel synergistic formulation based on the combination of linalool, methyl cinnamate and thymol has been developed applying low molecular weight chitosan dissolved in acetic acid solution. These compounds, known by their strong antimicrobial activity, are the major BACs of the essential oils obtained from the aromatic plants *Zanthoxylum alatum*, *Thymus vulgaris* and *Cananga odorata*. The nanoencapsulated formulation, characterized by an EE of 35–40% and a loading efficiency 6–7%, showed remarkable antifungal and anti-aflatoxin B1 protection both in vitro and in a food system (*Pennisetum glaucum*) without affecting the sensory properties [89]. Another case is the development of casein-based nanoencapsulation systems for delivery of epigallocatechin gallate and folic acid [8]. The obtained results are promising for the encapsulation of hydrophilic nutraceuticals. The delivery systems, dependent on pH, were characterized by a spherical shape and large amount of EE (85%).

Currently, other delivery systems for phenolic compounds are being developed. In this regard, lipid NPs such as nanoemulsions, nanoliposomes, solid lipid NPs (SLN) and nanostructured lipid carriers (NLC) have been used in the food industry. For instance, nanostructured lipid carriers (NLC) have been used and optimized by response-surface methodology to protect BACs from *Hibiscus sabdariffa* [90]. The impact of the emerging technology used for the extraction of phenolic compounds, microwave-assisted extraction (MAE) or pressurized liquid extraction (PLE), lipid and surfactant concentration on the average NPS, PDI and ZP were evaluated. MAE-NLC resulted in EE of 52.9 and 60% for quercetin and anthocyanins, respectively, and NPs characterized by a mean size of 470 ± 8 nm, PDI of 0.47 and ZP of −26.3 mV. These NPs retained 55% of total phenolic compounds (TPC), while PLE-NLC NPs resulted in higher retentions (73%) and EE (93 and 84% for quercetin and anthocyanins, respectively) with a lower NPs size and PDI but higher ZP (344 nm, 0.34 and −25.7 mV, respectively). These NLC showed additional favorable attributes compared to those found in other NPs, such as more loading capacity and longer physicochemical stability, due to the combination of solid and liquid lipids forming their matrix [91].

Total phenolics and anthocyanins from blueberry pomace were recently encapsulated after successfully developing a food-grade water-in-oil-in-water or double nanoemulsion system. Particularly, the influence of critical physical parameters (homogenization pressure, stirring speed and time) on the characteristics of double emulsions was evaluated [70]. The authors found that particle size, size dispersity, ZP and EE greatly impact the stability and functional performance of nanoemulsions (e.g., appearance, texture and bioavailability) and of the final products in which they are used [70]. Min et al. [7] nanoencapsulated resveratrol (a naturally occurring polyphenolic compound from the stilbenes family, mainly found in peanuts and grape skins) in trimethyl chitosan NPs cross-linked with tripolyphosphate and/or alginate to acquire controlled release and improved cellular uptake. The combination of both materials with alginate showed higher particle size (575 nm) and EE (56.9%), better distribution (0.339), and the effective controlled release of bioactive materials from chitosan NPs, probably due to the high molecular weight and viscous property of alginate.

Carotenoids are important phytonutrients, essential for the health since humans are unable to synthesize them in their bodies. These compounds are interesting functional ingredients in the human diet that also can be used as coloring agents in processing. Therefore, in addition to their immense importance in photosynthetic organisms as natural pigments, they are considered as valuable health-promotional compounds. Nevertheless, they are susceptible to environmental and processing stress while having low bioavailability and water solubility. Changes of their chemical structure are prone to influence of pH, light, oxygen and heat, limiting their pharmaceutical/food exploitation [92]. In this context, nanotechnology through lipid-based nanodelivery structures (nanoemulsions, nanoliposomes or surfactant-based nanocarriers and SLN) offered safe and positive options for the entrapment of carotenoids. As a result, an efficient controlled release is obtained, which would allow the expansion of its industrial applicability [93,94].

In this regard, dos Santos et al. [95] described a series of carotenoids delivery systems and the application of different techniques to determine the parameters that could restrict the effective use of nanoencapsulation technology in the pharmaceutical, cosmetic and food industry. NPs obtained from tomato carotenoid lycopene have been prepared and accepted as GRAS and were currently used as a food additive. They can be incorporated in baking products and in soft drinks to provide not only color, but also to promote health benefits [6]. It has been reported that tomato coproducts represent a valuable source for the extraction of potential natural food colorants, antioxidants or supplements with a variety of health benefits. However, as already mentioned, it is not easy to integrate carotenoids into various products due to their inherent instability. Hence, a novel study performed by Horuz and Belibağlı [39] evaluated the use of an electrospinning technique for the nanoencapsulation of carotenoids isolated from tomato peel into zein nanofibers to increase their antioxidant capacity and stability, showing the possibility of its promising use in food processing.

Bezerra et al. [92] evaluated the use of yellow passion fruit albedo flour, with important pectin content, as encapsulating component for the development of nanodispersions of carotenoid extract from *Spirulina* sp. LEB 18. Nanodispersions were characterized for their stability, antioxidant capacity, physicochemical features and retention of carotenoids after 60 days of storage at 4 °C. Concluding remark identified flour as a natural alternative to commercial polymers, containing the possibility of their introduction into several food formulations.

The nanoencapsulation of saffron hydrophilic apocarotenoids, as crocins and picrocrocin, using maltodextrin and spray-drying was also reported. The production and the characterization of the product stability were evaluated in the study by examining the influence of size and core: wall ratio on the product yield and the EE. Three were the core: wall weight ratios, 1:5, 1:10 and 1:20 (*w/w*) evaluated. The effect of the core: wall ratio on EE was similar for crocins and picrocrocin. Thus, as the ratio increases, there is a decrease in EE (≅54% vs. ≅80% and ≅56% vs. ≅82% in crocins and picrocrocin for 1:5 and 1:20, respectively). This behavior could be related to the fact that a thin layer of wall material is formed when a higher ratio is used due to the low content of wall material in the initial feed solution [96]. In addition, authors stated that thermal stability and bioaccessibility of these apocarotenoids was increased with nanoencapsulation [97]. Additionally, BACs of saffron (crocin, safranal and picrocrocin) were also encapsulated by using whey protein concentrate (WPC)-maltodextrin or WPC-pectin-maltodextrin applying water-in-oil-in-water multiple emulsions [98]. The best results were obtained with the use of emulsions made with WPC/pectin complex, which would improve the protection of these ingredients against gastrointestinal conditions.

### 5.2. Essential Oils and Fatty Acids

Plant essential oils (EOs) have notable antimicrobial efficacy; hence they are promising alternative for synthetic additives [80]. Kavaz et al. [99] described EOs as volatile plant secondary metabolites that have already been extensively used in medicine, cosmetics and food industry for their antimicrobial, antifungal and antioxidant activity. However, their application is still not completely effective due to some drawbacks (low water solubility, bioavailability, volatility and stability in the foods). The progress made in nanotechnology in recent years allows one to address these obstacles for use of EOs as preservatives.

The possible carrier agents for low doses of EOs are researched by industries in order to improve the quality of their final products. Actually, many applicable methods have been developed to encapsulate EOs and improve their stability [46]. Nanoemulsions, liposomes and solid-lipid NPs are currently used strategies for protection of plant BACs [3]. Furthermore, essential and fish oils can exert unpleasant flavors, which limits their direct inclusion into food formulations, due to their negative impact on the sensory quality. In this sense, nanoencapsulation is useful for masking unwanted flavors [69]. Considering all the above mentioned, nanoencapsulation is presented as a suitable technique for protecting EOs from evaporation and oxidation, showing prolonged activity for encapsulated BACs by a controlled liberation.

Regarding the antimicrobial activity of EOs, the encapsulation of clove essential oil (CEO), with eugenol as the main component, was carried out by emulsion/ionic gelation technique with chitosan to enhance the antifungal efficacy of the cited oil [36]. The nanoencapsulated CEO showed a higher effect against *Aspergillus niger*, isolated from spoiled pomegranate, in comparison with free oil. Therefore, CEO-chitosan NPs could be an interesting natural fungicide in agricultural and food technology. In the same way, *Cyperus articulates* EOs (CPEOs) loaded with chitosan NPs (CSNPs) can be suitable for food and pharmaceutical industries due to their antibacterial activity (*E. coli* and *S. aureus*) and physiochemical properties [99].

In respect of the technological application, a recent study evaluated the use of whey protein wall system to nanoencapsulate roasted coffee bean oil through nanospray drying process [23]. This product is considered as a substantial by-product obtained from the coffee industry, which is commonly used as flavor ingredient in food products. However, coffee oil is highly susceptible to oxidation with the consequent formation of undesirable flavors. Therefore, its use in nanoemulsion form would allow to improve its stability and to protect coffee oil constituents, probably related to the emulsifying properties of whey protein isolate and the lower oil-to-wall ratio used. Ghasemi et al. [14] studied the encapsulation of orange peel oil by pectin–whey–protein nanocomplexes. This oil is a valued flavoring agent in the food industry, but it is volatile under environmental conditions. Thus, applying nanoencapsulation technology by complexation method, where the core substance is protected with a complex of two different biopolymers, is optimal way to preserve and control the release of the oil.

Furthermore, a recent study evaluates the possibility of encapsulating fish oil with high content in ω-3 long chain polyunsaturated fatty acids in lecithin-oil mixture nanoliposomes. The aim was to evaluate its influence on the technological and sensory quality of fortified bread, since this oil is characterized by strong odor and easy oxidation. The results revealed that nanoencapsulation of fish oil exerted higher resistance to lipid oxidation and its incorporation into bread had no negative effects on both textural and sensory quality [75].

### 5.3. Vitamins

In recent years, the interest for vitamins has been growing due to their double roles as nutrients and disease control agents. In the context of functional foods, the healthy properties that liposoluble vitamins provide to foods have been extensively valued by the industry. Nevertheless, their sensitivity to oxidation and their low water solubility (for liposoluble vitamins) are a limiting factor for their efficacy. In this regard, nanoencapsulation again offers opportunities to enhance the bioavailability, shelf life and their controlled release [100].

Nanoencapsulation can protect many vitamins against a wide range of conditions and substances that could otherwise affect their integrity during food processing. As an example, David and Livney [83] used potato proteins as a natural material to protect and deliver vitamin D3 (VD) in beverages. VD-potato protein nanocomplexes had better results regarding the shelf life of the samples with reduced loss of vitamins. In addition, Abbasi et al. [101] encapsulated VD by whey protein isolates NPs and evaluated its stability for 7 days in the presence of air. They observed higher content of vitamin D in NPs than in control sample.

Chapeau et al. [102] investigated coassembly to bind lactoglobulin and lactoferrin as biocarriers of the vitamin B9, and concluded that natural food components have an important potential as carriers of functional ingredients. Other natural carrier materials are alginate and chitosan that were used by Azevedo et al. [103] for developing vitamin B2 NPs. Riboflavin was also encapsulated in food-grade water/oil/water double emulsions with a variety of lipid sources (chia oil, sunflower oil, olive oil or rendered pork backfat).

In the case of vitamin E (“tocopherol”), Hategekimana et al. [104] applied nanoemulsion technology to obtain nanocapsules made with octenyl succinic anhydride starches as emulsifiers and wall materials [105]. This vitamin E loaded-nanocapsules could be applied in the pharmaceutical and beverage sector. Dasgupta et al. [82] produced vitamin E nanoemulsions by edible mustard oil and Tween 80 as a surfactant. Double emulsions can be another way for nanoencapsulating BACs [106].

### 5.4. Peptides and Enzymes

There were not many studies about the nanoencapsulation of peptides and enzymes. Maherani et al. [107] successfully encapsulated natural dipeptide antioxidants in nanoliposomes, while Wen et al. [108] applied liposomes in the oral delivery of enzymes, proteins, flavors and antimicrobial substances. In one study, two-fold reduction for cheese elaboration was possible through the entrapment of proteolytic enzymes into liposomes without affecting texture and flavor [6]. Nisin, that is a polycyclic antibacterial peptide, was encapsulated and assayed for antimicrobial capacity in skimmed milk and in cheese inoculated with *Listeria monocytogenes* [77]. Free nisin served as a control while encapsulated nisin demonstrated better inhibitory properties in milk and cheese at refrigeration temperatures [109]. Likewise, Janes et al. [110] developed chitosan NPs of polypeptides, observing that the prepared particles presented improved uptake through mucosal cells.

Quinoa-based proteins can be considered a rich source of bioactive peptides to which they are attributed important health benefits and strong antioxidant and antimicrobial activities, what was reflected in its incorporation through nanoliposomes in fresh beef burgers [79]. This is also the case of fish skin peptides, marine byproducts and natural antioxidants, applied as biopreservative through lecithin nanoliposomes in pork patties [76].

### 5.5. Probiotics and Prebiotics

Another attractive trend reported by Alfadul and Elneshwy [111] is the nanoencapsulation of live probiotic microbes for the promotion of gastrointestinal health. They can be incorporated into different food and drink products, including fermented milk, cheese, yoghurt and fruit-based drinks. Particularly, the main genera of probiotic microorganisms preferred by the food industry are *Lactobacillus* and *Bifidobacterium*. However, the application of probiotics in food products is not an easy procedure, since several of these microorganisms might lose their viability before consumption (during processing and storage) or during digestion [43]. Therefore, it is important to keep in mind that when these compounds are applied for health improving purposes, it is required that a minimum of 10^6^ CFU/g of intestinal content of probiotic bacteria are present in the final food product [112]. That is why encapsulation could represent an interesting solution to this problem, avoiding the reduction of the living probiotic bacteria.

In the same way, the intake of prebiotics is being considered as a healthy habit since this group of ingredients (inulin, lactulose, fructooligosaccharides, galactooligosaccharides or the human milk oligosaccharides) has shown properties that contribute to the well-being of the consumers [113]. Among others, their beneficial effects include: control of cholesterol, cancer prevention, improvement of mineral bioavailability, constipation relief or treatment of diarrheas and allergies [114]. In this regard, the influence of galactooligosaccharides in the growth of encapsulated *Lactobacillus acidophilus* and *Lactobacillus casei* in alginate and chitosan microbeads was compared with inulin in yogurt and fruit juice [115]. The authors detected higher positive impact in probiotics viability when the galactooligosaccharides were incorporated. A few years ago, the positive effects of probiotics and prebiotics on human health, their incorporation in different food products, their possible synergistic interactions and the development of suitable encapsulation systems for their controlled and targeted release has been reviewed in details by Durazzo et al. [116].

## 6. Critical Conclusions and Prospects

Functional food market expanded over the decades due to increased consumers demand for natural, nutritional and healthy food products. Similarly, there are innumerable demands for reducing the levels of synthetic preservatives, looking for improved quality organoleptic features [18]. The knowledge supplied by researchers and industrial food professionals plays a key role in order to perform this leap forward. The increased funding to promising methods, scaling up of nanoencapsulation to industrial levels is expected to increase in upcoming years. Similarly, further investment in advanced and innovative food processing technologies is expected.

Nanoencapsulation-based technologies are unique and current, their operation in both, the food and pharmaceutical areas results in new opportunities for commercialization. In particular, their application involves the production of nanostructured food ingredients, which allow the improvement of the solubility, stability flavor, color and texture of foods during processing and storage. For instance, encapsulation is a valid option to hide unpleasant flavors or aromas. Additionally, it increases the bioavailability and controlled release of the different nutrients in food (protecting bioactive target compounds from light, oxygen or pH variations) while at the same time volatility of encapsulated compounds decreases. Therefore, food businesses can benefit from incorporating NPs to their novel food and beverage products [6,41]. Considering the challenges, first it would be advisable to decrease the costs and complexity of nanoencapsulation-based systems. Moreover, it is necessary to establish the interactions between the bioactive ingredients and the multiple encapsulating carriers in food systems.

Since a great variety of novel materials are continuously added to foods, many countries established substantial scientific platforms to control food safety and provide risk assessments [117]. It should be clear that there is still limited data on the toxicological repercussions and ethical issues of nanomaterials, so they must be rigorously tested before being commercialized. This should be done by appropriate in vivo models, which will allow the establishment of legal regulations to protect consumers’ safety [118]. With this in mind, society and industry can benefit from novel applications of nanotechnology in food production, protecting the safety, the health and the environment [3,6,15]. From another point of view, nanotechnology can also play a notable role in the development of programmable food, an original futuristic concept that promise consumers to obtain their food with the desired nutritional, color, flavor and texture characteristics [3]. Overall, there is an optimistic attitude and perspective to fully implement and introduce nanoencapsulation in the food industry, which seems to expand in near future and improve the standard of living of the people.

## Figures and Tables

**Table 1:** Summary of habitual food BACs (core substances) that are susceptible to be nanoencapsulated.

BACs	Application/s of Their Encapsulation	Examples
Phenolic compounds	Protection, improvement of their antioxidant and other functional activities, incorporation of additional positive properties, target delivery	Quercetin, tea catechins, folic acid, thymol, resveratrol, anthocyanins
Carotenoids	Stabilization, efficient controlled release, expansion of their industrial applications (as colorants and antioxidants)	Tomato lycopene, crocins
Essential fatty acids	Stabilization, better solubility, decrease volatility, use of lower doses, favorable impact on the sensory quality of the final product	DHA, linolenic acid,
Vitamins	Protection to oxidation	Vitamin D3, vitamin B9, vitamin B2, riboflavin, thiamine
Peptides and enzymes	Improved antimicrobial and/or antioxidant activity, better absorption	natural dipeptides, nisin
Probiotics and prebiotics	Increment of viability, promotion of gastrointestinal health, suitable inclusion in the food product	*L. casei*, *L. reuteri*, *B. bifidum*, *B. breve*, *S. thermophilus*, fructooligosaccharides galactooligosaccharides, lactulose
Others	Stabilization, controlled release, improved homogeneity, flavor, taste and/or texture of the food system	Minerals, colorants, flavors, buffers, micronutrients

**Table 2:** Commonly used wall materials for encapsulation of food components and representative characteristics to bear in mind.

Carbohydrates	Proteins	Fat and Waxes	Polymers
Chitosan	Whey protein	Hydrogenated vegetable oils	Polyglycolides
Pectin	Sodium caseinate	Bee wax	Polylactides
Maltodextrin	Soy proteins	Lecithin	Polyethylene glycol
Alginates	Gelatin	Medium chain triglycerides	Polyorthoesters
Cyclodextrins	Caseins		Polyanhydrides
Gum Arabic	Zein		Polyacrylamide
Cellulose derivatives			Polycaprolactone
Modified starches			Polyacrylic acid
Carrageenan			Polyvinyl alcohol
Properties that should be considered:	Solubility, edibility, film-forming ability, emulsification, viscosity, degree of crystallinity, compatibility with the core compounds, cost, taste and flavor		

**Table 4:** Application of nanoencapsulated bioactive ingredients into food products.

BACs	Nanocarriers	Technique	Goal	Food Product	Dose Used	Storage	Main Effects	Ref.
Chlorogenic acids	β-cyclodextrin	Nano-complexation	Phenolic compounds Supplementation on aroma volatile profile	Bread, cookies, caramel cottage cheese, nutty filling, and mushroom or meat stuffing	237.7 mg/g	–	Limited formation of preferred aroma volatiles	[74]
Fish oils	Lecithin sunflower oil	Nanoliposomes	Improvement of the nutritional value	Bread	5% (*w*/*w*)	25 days	Oxidative stability against lipid oxidation. Positive effects on technological and sensory properties	[75]
		Nanoliposomes Mozafari method	Fortification	Yogurt	15 mL/100 g	21 days	High DHA and EPA contents. Avoid strong odors and rapid deterioration. Good sensory attributes. ↓ acidity, peroxide values and syneresis	[54]
Fish skin peptide	Lecithin	Nanoliposomes Ultrasonication	Preservative and vehicle for entrapping fishy smell	Pork patties	0.1%, 0.5%, 1%, 2%, and 3% (*w*/*w*)	14 days at 4 °C	Inhibitory effect on lipid oxidation Dose dependent effect	[76]
Lupulon xanthohumol		Nanoliposomes Ultrasonication	Replacement of nitrite	Cooked beef-sausage	50, 100, 150 and 200 ppm	30 days at 4 °C	Prevention of microbial growth and lipid oxidation. Addition of nanoliposomes has not impaired sensory properties	[68]
Nisin and garlic extract	Phosphatidylcholine and oleic acid	Nanoliposomes Thin-film hydration/Ultrasound	Antimicrobial agent	Whole UHT milk	–	25 days at 7 °C	Improved the activity of nisin against Gram-negative bacteria. Protected the antioxidant activity and masked undesirable odors	[77]
Nisin and lysozyme	Phosphatidylcholine-pectin/polygalacturonic acid	Nanoliposomes	Natural antimicrobials	Whole or skim UHT milk	0.05–0.1%	25 days	Inhibition of *Listeria monocytogens* and *Salmonella Enteritidis*	[50]
Olive leaf phenolics-oleuropein	Lecithin cholesterol	Nanoliposomes Ethanol injection	Functional food product	Yogurt	0.7% phenolics/g nanoliposome	21 days	Antioxidant activity. Good sensory attributes and syneresis	[78]
Prickly pears peel fruit	Sodium alginate-chitosan	Nanoencapsulates	Preservative	Guava juice	0.1%	120 days	Ability to withstand the high temperatures and pasteurization	[13]
Quinoa peptide	Soy phosphatidylcholine and cholesterol	Nanoliposomes	Natural food preservative	Fresh beef burgers	3 and 5 mg/mL	12 days at 2–4 °C	Antimicrobial and antioxidant properties	[79]
*Thymus capitatus* EO	Aqueous SDS/soybean oil	Nanoemulsion Microfluidizer	Antimicrobial agent	Semi skimmed UHT milk	2 mg/mL	24 h at 37 °C	Improving physicochemical and microbial (*S. aureus*) quality of milk	[80]
α-Tocopherol	Canola oil and Tween 80	Nanoliposomes Magnetic stirring/Ultrasound	Antioxidant agent	Fish sausages	250, 500 mg/kg	16 days at 4 °C	Lower spoilage rate. Delayed lipid oxidation during storage	[81]
Vitamin E	Edible mustard oil-surfactant Tween-80	Nanoemulsion Wash-out method	Health supplement. Antioxidant	Mango juice	1:9, 2:8, 3:7, 4:6 and 5:5	24 h at 37 °C	Reduction in microbial growth	[82]
Vitamin D_3_	Lecithin	Phase-inversion temperature method	Fortification	Milk	4% and 8% (w/w)	–	Protect vitamin against acidic conditions. Good acceptability	[67]
	Potato proteins	Protein nanocomplexes	Promotion of human health	Clear beverages solutions	10, 25, 50, 75 and 100 μg/mL	Three weeks	Protection during pasteurization and shelf life	[83]

## Data Availability

The data presented in this study are available on request from the corresponding authors.
